# Survey of knowledge, attitude and practice of healthcare professionals on dengue transmission, diagnosis and clinical classification

**DOI:** 10.1186/s12879-021-06816-y

**Published:** 2021-11-02

**Authors:** Hoang Thi Nam Giang, Ahmed M. Sayed, Thao Dang, Somia Iqtadar, Nguyen Minh Tuan, Nguyen Tuan Khiem, Do Chau Viet, Tran Thi Kim Van, Nguyen Thanh Phuoc, Tran Thi Kim Dung, Esraa Ahmed Elhalwagy, Le Huu Linh Vien, Nguyen Minh Triet, Nguyen Thanh Tong, Do Hong Son, Lieu Chi Hung, Dong Thi Hoai Tam, Kenji Hirayama, Nguyen Tien Huy

**Affiliations:** 1 School of Medicine and Pharmacy, The University of Danang, Danang, 550000 Vietnam; 2 Faculty of Pharmacy, Al-Azhar University, Cairo, Egypt; 3 Department of Internal Medicine, Texas Tech University Health Science Center at the Permian Basin, Odessa, TX USA; 4 Dengue Expert Advisory Group Punjab, King Edward Medical University, Lahore, Pakistan; 5 Children’s Hospital 1, Ho Chi Minh, Vietnam; 6 Pham Ngoc Thach University of Medicine, Ho Chi Minh, 70000 Vietnam; 7 Children’s Hospital 2, Ho Chi Minh, Vietnam; 8 Cao Van Chi Hospital, Tay Ninh, Vietnam; 9 General Hospital of Agricultural, Ha Noi, Vietnam; 10 Faculty of Medicine, Tanta University, Tanta, Egypt; 11 The Le Ngoc Tung Hospital, Tay Ninh, Vietnam; 12 Thanh Hien Pharmacy, Tay Ninh, Vietnam; 13 The Tay Ninh General Hospital, Tay Ninh, Vietnam; 14 Department of Infectious Diseases, Faculty of Medicine, University of Medicine and Pharmacy at Ho Chi Minh City, Ho Chi Minh City, 70000 Vietnam; 15 Department of Immunogenetics, Institute of Tropical Medicine (NEKKEN), School of Tropical Medicine and Global Health, Nagasaki University, Nagasaki, Japan; 16 School of Tropical Medicine and Global Health, Nagasaki University, Nagasaki, 852-8523 Japan

**Keywords:** Classification, Dengue, Warning signs, Definition

## Abstract

**Background:**

To investigate the knowledge, attitudes, and practices of the healthcare professionals (HCPs) including physicians and nurses regarding dengue transmission, diagnosis and clinical classification using the warning signs of World Health Organization (WHO) 2009 classification.

**Results:**

Out of 471 respondents from three countries, 80.9% of physicians and 74% of nurses did not receive previous training regarding the dengue infection. The majority of respondents could identify the primary dengue vector (86%), while only a third of HCPs knew the biting time of dengue mosquitoes. Only half of our respondents knew about immunity induced by serotypes; Moreover, half of our participants could determine the diagnostic tests. On the other hand, about 90% of the respondents took responsibility for talking to the patients about preventive measures. Our respondents also showed wide variations in definition of warning signs listed in the WHO 2009 classification. Multivariate analysis linked the impact of different cofactors including prior training on dengue infection, type of profession, frequency of taking care of dengue patients and country on how HCPs defined these warning signs.

**Conclusions:**

This study could declare the variation in employing the warning signs listed in the WHO 2009 classification. We have figured that most of the HCPs did not take prior training on the dengue viral infection; Also, we found gaps in the knowledge regarding various topics in dengue fever. This paper recommends the gathering of efforts to establish the proper knowledge of dengue infection and the warning signs listed by the WHO.

**Supplementary Information:**

The online version contains supplementary material available at 10.1186/s12879-021-06816-y.

## Introduction

Dengue is a mosquito-borne viral disease that encompasses 129 countries around the world [1–3]. Half of the global population stands at risk of dengue infection and the other half remains in dengue-endemic countries [4–6]. The world witnesses more than five million dengue infection reported from World Health Organization (WHO) regions in 2019, which is about tenfold higher compared to that in 2000 [3]. All six regions of WHO were affected, however three most seriously affected regions are South-East Asia, Western Pacific Regions, and recently the Region of the Americas.

The infection of dengue starts with a bite of a female mosquito primarily of the species *Aedes aegypti* and, to a lesser extent, *Aedes albopictus*, *Aedes polynesiensis*, and *Aedes scutellaris.* There are four dengue serotypes: DEN-1, DEN-2, DEN-3, and DEN-4, and symptoms typically appear in four to seven days going through three phases: febrile, critical, and recovery [7]. Most infections by any of these serotypes result in a self-limiting fever; however, age, genetic predisposition, and background immunity can result in different clinical appearances, the most severe of which causes hemodynamic compromise due to plasma leakage [8, 9]. In 2009, WHO classified dengue into three categories: dengue without warning signs, dengue with warning signs and severe dengue to optimize clinical management since the strict application of the 1997 system missed the more severe manifestations [7]. Since a modest proportion of infected patient progress to the severe forms of dengue, it becomes more important than ever for healthcare professionals (HCPs) to recognize the warning signs in clinical practice.

Although the widely accepted WHO 2009 system was more sensitive to the severe manifestations of dengue compared to the 1997 system, it is lacking a clear definition of the warning signs [10]. Healthcare providers and researchers employed different criteria to define the warning signs, which led to diverse application of the guideline for the diagnosis and treatment of dengue patients. Therefore, there is an urgent need for the clear definitions of the warning signs and the severities in order to prevent unnecessary hospitalizations and to optimize triage and management [10–13]. Few studies have investigated the definitions used by healthcare providers on the field and their subsequent outcomes. This study was conducted in eight hospitals in three different countries to investigate knowledge, attitudes, and practices of the healthcare professionals regarding dengue transmission, diagnosis and clinical classification using the warning signs of WHO 2009 classification.

## Methods

### Study location

This study was conducted in three countries, including eight hospitals, six of them in the southern region of Vietnam, one in Egypt and one in Pakistan. The survey was conducted from April 2018 to June 2018. Vietnam is a tropical country with high burden of suffering dengue infection [3]. WHO 2009 classification has been widely used in Vietnam since the Ministry of Health issued the decision 458/QD-BYT in 2011. On the other hand, Pakistan has experienced an epidemic of dengue fever since 2010 but continues to use WHO 1997 classification. In another context, dengue infection has re-emerged in Egypt since 2011.

### Study population and design

This study is a cross-sectional survey using a self-administered questionnaire designed by physician-researchers and dengue specialists. The first draft of questionnaire was developed based on literature review and questionnaire of previously published studies [12, 14–16]. Three infectious disease specialists thoroughly reviewed the questionnaire. Based on specialist’ comments, revisions were carried out with regard to content, arrangement and structure of questionnaire. Then, two pilot surveys were distributed to students of the Online Research Club (www.onlineresearchclub.org) through google forms to check the reliability of the questionnaire. The primary version of the questionnaire was English. Forward-translations and back-translations were used to translate the instrument into Vietnamese as recommended by WHO [17]. Vietnamese questionnaire was used in Vietnam, and English questionnaire was used in Egypt and Pakistan. The developed questions were intended to gauge the general dengue knowledge of the physicians and nurses (transmission, control measures), their recognition of the dengue warning signs based on the WHO 2009 classification, as well as their demographic information. The study populations are the doctors and nurses serving in either the pediatric or adult unit of the eight hospitals, located in Vietnam, Egypt, or Pakistan.

### Data collection

After getting approval from the hospitals, our data collectors distributed the questionnaire to all nurses and doctors compatible with our inclusion criteria. Consent was obtained in the form of a question. In total, we sent the questionnaire to 521 healthcare professionals and received 471 completed questionnaires, corresponding a response rate of 90.4%. The reason for refusal was not revealed in all cases, however, most of them were too busy to spend time on filling the questionnaire. Additionally, healthcare professionals were informed that their participation is voluntary, and that there no incentives for participating.

### Statistical analysis

Percentages of doctors and nurses choosing a specific answer for a question involving general knowledge or warning signs were obtained. Chi-squared test was performed to compare proportions of categorical variables. A p-value less than 0.05 was considered statistically significant. Multivariable logistic regression analysis was then used to assess the impact of the profession (physician or nurse), country of participants (Vietnam or Egypt or Pakistan), prior training on dengue infection, as well as the frequency of treating dengue patients on participants’ definition of warning signs. The numbers and proportions of the doctors and nurses using either one, two, three, four, or more than five warning signs of the WHO 2009 classification were also calculated to show the variations of healthcare workers defining these signs. Statistical analysis was then conducted using R Statistical Language (R Foundation for Statistical Computing, Vienna, Austria).

## Results

### Demographic information

Among the 471 respondents, 186 were physicians and 285 were nurses. More than half of the physicians were female (53.2%), and most of the nurses were female (83.2%). Moreover, 80.9% of physicians and 74.0% of nurses have received prior training on dengue fever. On average, physicians and nurses had around six years of experience in taking care of dengue patients. Nearly half of physicians (47.5%) and about a third of nurses (35.4%) chose either “very often” or “often” in terms of frequency of taking care of dengue patient. Additionally, more than 60% of physicians and nurses practiced in a provincial hospital (Table 1).

**Table 1:** Demographic information of participants

Characteristics		Doctors (N = 186)	Nurses (N = 285)
Age, mean—year (SD)		34.3 (8.9)	31.8 (7.3)
Male—no. (%)		87 (46.8)	48 (16.8)
Received training on dengue fever—no. (%)		148 (80.9)	208 (74.0)
Year of experience taking care of dengue patients, mean—year (SD)		5.3 (5.9)	6.7 (6.6)
Practice site—no. (%)	Community health center	1 (0.6)	1 (0.4)
	District hospital	18 (10.0)	50 (18.2)
	Provincial hospital	110 (61.1)	177 (64.6)
	Tertiary hospital	51 (28.3)	46 (16.8)
Country—no. (%)	Egypt	11 (5.9)	10 (3.5)
	Pakistan	27 (14.5)	–
	Viet Nam	148 (79.6)	275 (96.5)
Frequency of taking care of dengue patient—no. (%)	Very often (> 10 cases/week)	42 (23.3)	47 (17.1)
	Often (1–10 cases/week)	44 (24.2)	50 (18.3)
	Sometimes (1–10 cases/month)	59 (32.6)	109 (39.8)
	Seldom (1–10 cases/year)	36 (19.9)	68 (24.8)

### Answer to dengue queries among healthcare professionals (HCPs)

Most HCPs (86.0%) correctly identified the primary vector of dengue infection is Aedes aegypti (Table 2). A third of HCPs (31.6%) correctly answered the biting time of mosquito is during the daytime. For the dengue transmission, 90.9% of HCPs were familiar with the human-to-mosquito-to-human cycle of transmission; however, most of them failed to acknowledge rare events, including blood transfusion, organ transplantation, and transmission of dengue virus from mother to fetus. Furthermore, half of the HCPs (51.6%) were aware of different serotypes of dengue and the lifelong immunity bestowed by infection of any one of them. Most of the HCPs (79.8%) said that patients might be an intermediate host that transmit the viruses to the mosquito two days before the onset of fever and until the febrile state.

**Table 2:** The proportion of answers for knowledge question of healthcare professionals

Category of question		Proportion of respondents			
		Total (N = 471) n (%)	Doctor (N = 186) n (%)	Nurse (N = 285) n (%)	P
Dengue viruses are transmitted to humans through the bites of infective females of	*Aedes aegypti*	405 (86.0)	180 (96.8)	225 (78.9)	< 0.0001
	*Non-Aedes aegypti*	94 (19.9)	47 (25.3)	47 (16.6)	0.02
Transmission route	Human to human via a bite of an infected mosquito	428 (90.9)	168 (90.3)	260 (91.2)	0.7
	Mother to fetus or neonate at parturition	49 (10.4)	28 (15.1)	21 (7.4)	0.007
	Blood transfusion and Organ transplantation	34 (7.2)	25 (13.4)	9 (3.2)	< 0.0001
	Needle stick	15 (3.2)	7 (3.8)	8 (2.8)	0.5
	Sexual intercourse	3 (0.6)	2 (1.1)	1 (0.4)	0.3
Time when the dengue mosquitoes likely to feed/bite. (Day time)		149 (31.6)	68 (36.6)	81 (28.4)	0.06
There are different serotypes of dengue and being infected with one of them gives lifelong immunity to that serotype. (True)		243 (51.6)	104 (55.9)	139 (48.8)	0.1
The common test that takes shorter time to diagnose acute recent infection of dengue virus (NS1 strip test)		254 (53.9)	97 (52.2)	157 (55.1)	0.5
The specific serological tool for the determination of dengue antibodies (IgM ELISA)		202 (42.9)	86 (46.2)	116 (40.7)	0.2

There were 53.9% of HCPs able to identify that the NS1 strip test is common test that takes shorter duration of time in order to diagnose recent acute infection. Regarding serological test for the detection of dengue antibodies, 42.9% of HCPs selected IgM ELISA. Higher proportion of physicians identified IgM ELISA as specific serological diagnostic tool compared to nurses, however, the difference was not statistically significant (p = 0.2).

Multivariable logistic regression analysis showed that Vietnamese HCPs acquired better knowledge compared to HCPs in Egypt and Pakistan (p = 0.01). There was a significant difference between the overall performance of physicians and nurses (p = 0.047) (Additional file 1: Table S1). Physicians were better than nurses in identifying vectors of the dengue virus (p < 0.0001) and rare events of dengue transmission, including mother to the fetus (p < 0.007) and blood transfusion or organ transplantation (p < 0.0001).

### Attitudes and practices regarding dengue disease

According to Additional file 1: Table S2, 90.2% of HCPs “agreed” or “strongly agreed” on the responsibility to talk to patient’ families about dengue virus transmission and prevention. There were 73.8% of HCPs “always” or “often” speak to patient’ families about the cause of dengue. When 75.9% of HCPs “always” or “often” recommend preventive measures to stop mosquito bites at home, 73.1% of HCPs “always” or “often” advise protective measures against the spread of dengue for other people living in the same house with the patient.

It is observed that HCPs, who “agree” or “strongly agree” on their responsibility of informing the patients about dengue transmission and prevention, were more likely to inform the patient about the cause of the disease (adjusted Odds Ratio (aOR) = 4.88; 95% CI = 2.55–9.35), preventive measures against mosquito bites (aOR = 5.25; 95% CI = 2.71–10.18) as well as on preventive measures against the spread of dengue fever to other people in the household (aOR = 2.49; 95% CI = 1.28–4.84).

It is also observed that physicians were less likely to talk to patients about preventive measures against mosquito bites at home compared to nurses (aOR = 0.51; 95% CI = 0.31–0.84). Furthermore, HCPs, who deal with more than one dengue case per week, were more frequent to advise with protective measures to deter the spread of dengue fever for patients' family members (aOR = 2.12; 95% CI = 1.32–3.39).

Finally, it is observed that there were 47 (10.3%) HCPs who would give IV fluid or Ibuprofen for fever in dengue patients (8.8% physicians and 11.2% nurse, p = 0.4). Multivariable logistic regression analysis and data are shown in Additional file 1: Tables S2, S3.

### Knowledge of WHO 2009 classification of dengue cases and variation in the definition of warning signs

37.8% of HCPs chose correctly that at least one warning sign is needed to establish “dengue with warning signs” according to WHO 2009 standards. Our data showed that physicians were more likely than nurses to do so but the difference was not statistically significant.

Our respondents were provided varied definitions of the dengue warning signs: abdominal pain, persistent vomiting, mucosal bleeding, plasma leakage, liver enlargement, hemoconcentration, decrease in plate count, and lethargy. All definitions of each warning sign provided in the questionnaire were chosen by the doctors and nurses (Tables 3, 4). Nearly half of HCPs used more than one definition to define each warning sign, 229 HCPs (48.7%) chose five different definitions for mucosal bleeding and 87 HCPs (18.9%) chose five different definitions for plasma leakage. On the other hand, nearly a quarter and a fourth of HCPs answered “do not know/not sure” when asked about the definition of the rapid decrease in platelet count and lethargy. Proportion of nurses answered to definition of hemoconcentration “do not know/not sure” was significantly higher than doctors (p = 0.0003).

**Table 3:** Definition of warning signs

Warning signs	Physicians (186) Frequency (Percent)	Nurses (285) Frequency (Percent)	P-value
Abdominal pain			
Abdominal tenderness	85 (45.7)	93 (32.6)	0.005
Continuous abdominal pain	93 (50.0)	107 (37.5)	0.007
Increasing pain	70 (37.6)	132 (46.3)	0.06
Do not know/Not sure	7 (3.8)	47 (16.5)	< 0.0001
Persistent vomiting			
≥ 6 episodes of vomiting in 24 h	37 (19.9)	51 (17.9)	0.6
≥ 5 episodes of vomiting in 24 h	29 (15.6)	28 (9.8)	0.06
≥ 3 episodes of vomiting in 1 h	52 (28.0)	82 (28.8)	0.9
Vomiting for ≥ 2 consecutive days	46 (27.4)	103 (36.1)	0.05
Vomiting with signs of dehydration on physical examination	55 (29.6)	79 (27.7)	0.7
Vomiting whenever eating/drinking	45 (24.2)	55 (19.3)	0.2
Do not know/Not sure	17 (9.1)	40 (14.0)	0.1
Mucosal bleeding			
Epistaxis	149 (80.1)	199 (69.8)	0.01
Gingival bleeding	149 (80.1)	237 (83.2)	0.4
Hematemesis, Melena	123 (66.1)	172 (60.4)	0.2
Hemoptysis	58 (31.2)	46 (16.1)	0.0001
Ear bleeding	42 (22.6)	36 (12.6)	0.004
Hematuria	94 (50.5)	72 (25.3)	< 0.0001
Menorrhagia, metrorrhagia, vaginal bleeding	116 (62.4)	152 (53.3)	
Petechia, purpura, ecchymosis, bruises	72 (38.7)	154 (54.0)	0.05
Conjunctival, subconjunctival, retinal hemorrhages	108 (58.1)	138 (48.4)	0.03
Plasma leakage			
Hemoconcentration	162 (87.1)	218 (76.5)	0.005
Pleural effusion	130 (69.9)	157 (55.1)	0.001
Gall bladder thickening	95 (51.1)	64 (22.5)	< 0.0001
Ascites	101 (54.3)	97 (34.0)	< 0.0001
Edema (face and extremities)	64 (34.4)	66 (23.2)	0.009
Free fluids around the urinary bladder	–	–	
Do not know/Not sure	4 (2.2)	26 (9.1)	0.003
Liver enlargement			
Liver edge palpated more than 2 cm below the costal margin	107 (57.5)	129 (45.3)	0.009
Liver edge palpated below the costal margin	33 (17.7)	81 (28.4)	0.008
Painful hepatomegaly	120 (64.5)	151 (53.0)	0.01
Depend on each case	27 (14.5)	70 (24.6)	0.008
Hemoconcentration			
Increasing in hematocrit by more than 20% from the baseline	133 (72.7)	158 (57.5)	0.0008
Increasing in hematocrit by more than 15% from the baseline	15 (8.2)	25 (9.1)	0.7
Using a cutoff value for hematocrit that is adjusted for gender	11 (6.0)	12 (4.4)	0.4
Using a non-adjusted cutoff value of hematocrit > 48%, with no discrimination between males and females	10 (5.5)	25 (9.1)	0.1
Do not know/Not sure	14 (7.7)	55 (20.0)	0.0003
Rapid decrease in platelet count			
< 20.000 mm^3^ (1)	17 (9.4)	19 (7.0)	0.3
< 50.000 mm^3^ (2)	43 (23.9)	36 (13.2)	0.003
< 100.000/ mm^3^ (3)	78 (43.3)	151 (55.5)	0.009
< 150.000/ mm^3^ (4)	4 (2.2)	12 (4.4)	0.2
Do not know/Not sure	38 (21.1)	54 (19.9)	0.7
Lethargy			
Alteration of consciousness and/or Glasgow score < 15 or Blantyre score less than 5	37 (20.2)	51 (18.2)	0.6
Drowsiness and/or irritability	92 (50.3)	157 (56.1)	0.2
Do not know/Not sure	54 (29.5)	72 (25.7)	0.3

**Table 4:** The variation in the number of warning signs that doctors and nurse use to define warning sign

Number of criteria (definition) among the listed definition used by HCPs	Abdominal pain		Persistent vomiting		Mucosal bleeding		Plasma leakage		Liver enlargement	
	Doctor (n = 185)	Nurse (n = 284)	Doctor (n = 181)	Nurse (n = 281)	Doctor (n = 185)	Nurse (n = 285)	Doctor (n = 184)	Nurse (n = 277)	Doctor (n = 184)	Nurse (n = 280)
One definition	92 (49.7)	125 (44.1)	92 (50.8)	145 (51.6)	25 (13.5)	47 (16.5)	42 (22.8)	88 (31.8)	91 (49.4)	122 (43.6)
Two definitions	46 (24.9)	66 (23.2)	45 (24.9)	63 (22.4)	6 (3.2)	16 (5.6)	14 (7.6)	43 (15.5)	63 (34.2)	63 (22.5)
Three definitions	30 (16.2)	29 (10.2)	17 (9.4)	15 (5.3)	20 (10.8)	39 (13.7)	34 (18.4)	54 (19.5)	21 (11.4)	40 (14.3)
Four definitions	7 (3.8)	5 (1.8)	9 (5.0)	8 (2.8)	24 (13.0)	56 (19.6)	41 (22.3)	28 (10.1)	2 (1.1)	12 (4.3)
≥ 5 definitions	3 (1.6)	12 (4.2)	1 (0.5)	10 (3.6)	106 (57.3)	123 (43.2)	49 (26.6)	38 (13.7)	1 (0.5)	5 (1.8)
Do not know/Not sure (6)	7 (3.8)	47 (16.5)	17 (9.4)	40 (14.2)	4 (2.2)	4 (1.4)	4 (2.2)	26 (9.4)	6 (3.3)	38 (13.6)

Sign includes: 1 = Abdominal tenderness; 2 = Diffuse pain; 3 = Continuous (not intermittent) abdominal pain; 4 = Intensive abdominal pain; 5 = Increasing abdominal pain; 6 = Do not know/Not sure; 7 = Other

**Fig. 1:**
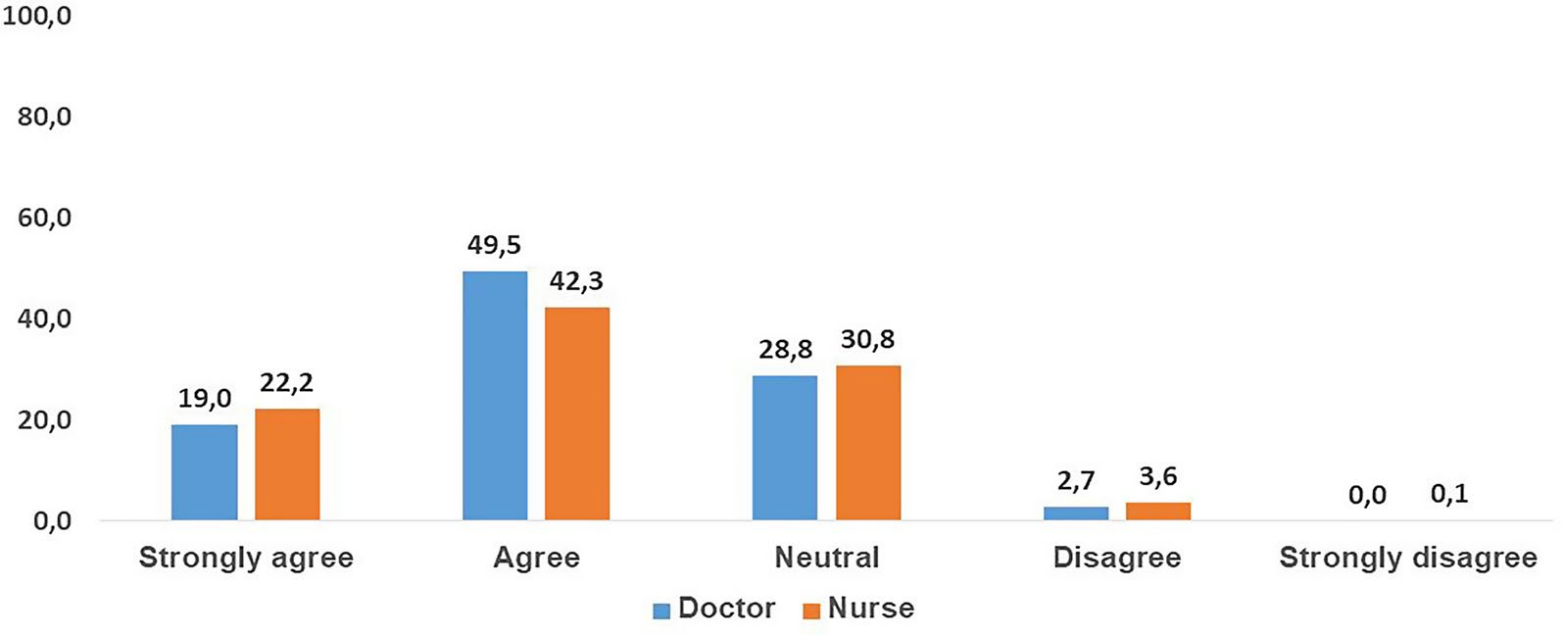
WHO 2009 Classification of dengue should be more precise or modified

Multivariable analysis showed a significant impact of different co-factors including the profession of HCPs (physician or nurse), country of HCPs, prior training on dengue fever, and frequency of taking care of dengue patients—on how HCPs defined warning signs (Additional file 1: Table S4). In responding to whether or not the WHO 2009 classification of dengue should be more precise or modified, 306 HCPs (66.1%) “agree” or “strongly agree,” 139 (30.0%) are “neutral,” 18 (3.9%) “disagree” or “strongly disagree” (Fig. 1).

Only 92 (49.7%) physicians and 125 (44.1%) nurses defined abdominal pain using one of five listed definitions, while the remaining HCPs used two or more definitions for this sign. For instance, each of these criteria: abdominal tenderness, and continuous abdominal pain were used by around two-fifth of the HCPs for the definition of abdominal pain: 37.8% and 42.5% respectively.

Regarding to the persistent vomiting, almost half of the HCPs (51.3%) used one definition, and the remaining used two or more definitions (48.3%). Each of these criteria for persistent vomiting: ≥ 3 episodes of vomiting in one hour, vomiting with a sign of dehydration on physical exam, vomiting whenever eating or drinking, was observed to be used by 28.4%, 28.4%, and 21.2% of HCPs respectively. The other definitions were chosen in lower proportion as seen in Table 4.

Fifteen percent (15%) of HCPs defined mucosal bleeding by one of nine definitions, and most HCPs used two or more criteria to define this warning sign. There were 78.9% of HCPs who specified that epistaxis as mucosal bleeding, 82.0% chose gingival bleeding, 62.6% answered hematemesis, melena was 62.6%, and finally 56.9% considered menorrhagia, metrorrhagia, vaginal bleeding as mucosal bleeding.

For plasma leakage, less than one-third of HCPs used one of six criteria, including hemoconcentration, pleural effusion, ascites, and gallbladder thickening to define plasma leakage. There were 22.8% of doctors who used one of six criteria, while the proportion of nurses who used one of six criteria were 31.8%. The difference was statistical significance (p = 0.03). The most common criteria that HCPs used for defining this warning sign were hemoconcentration (80.7%) and pleural effusion (60.9%). Around half of the HCPs considered liver edge palpated more than two centimeters below the costal margin as liver enlargement, while 59% of HCPs also used painful hepatomegaly. Hematocrit level increased more than 20% from the patient’s baseline value was cut-off levels chosen by 63.5% of HCPs to define hemoconcentration, while 8.7% used 15% from hematocrit baseline as criteria. 5% of HCPs used a cutoff value that adjusted for gender to define an increased hematocrit level. In contrast, 7.6% of HCPs using a non-adjusted cutoff value of hematocrit over 48%, with no discrimination between males and females. The remaining 15.1% of HCPs do not know/not sure how to define this sign.

Finally, platelet count < 100.000 mm^3^ was used for defining a rapid decrease in platelet count by approximately 50% of physicians and nurses; however, one-third of HCPs answered that they do not know/not sure. These variable definitions are summarized in Table 4 and Additional file 1: Tables S1–S4.

## Discussion

In 2009, the WHO published a classification of dengue cases, which was attributed to several benefits shown in literature [18]. One of those values was the considerable sensitivity in capturing the severe case [10, 19, 20]. Moreover, a study proved that this classification was easy to apply with its user-friendliness to healthcare providers [10]. Nevertheless, the WHO 2009 classification listed seven warning signs of dengue without providing a precise definition of these signs. These lead to the different application of these signs for the diagnosis and treatment of dengue patients between healthcare personnel.

A previous systematic review showed the wide variation of the definitions and cutoff values used by researchers to classify dengue patients [11]. Likewise, the current study found a wide variation in defining the warning signs among physicians and nurses in clinical settings. Of these seven warning signs, the HCPs have not agreed on any signs, even with the definition of liver enlargement, which was predefined in WHO 2009 classification. This variation may be linked to the local modification of the guidelines, adoption of national guidelines, lack of proper training, and variation in clinical practice in different regions.

A multi-center study in 18 countries concluded that the variations in the definition for warning signs could be observed in different regions [10]. Therefore, guideline adaptation at the local level is needed. Some HCPs denounce the WHO 2009 classification-related symptoms for being vague and associated with another disease [10]. Consequently, if the warning sign does not have a precise definition, the hospitalization rates might rise significantly; therefore, the definition and usefulness of warning signs in different regions, countries and levels of healthcare should be prospectively investigated.

Our results could link the resulting inconsistent definition of the warning signs to the specialties of HCPs (physicians or nurses), country of HCPs, prior received training on dengue fever, and frequency of taking care of dengue patients. However, there are limitations to this study. Because this study is a self-administered survey with given answer choices, the knowledge gap may not be sufficiently addressed compared to a blank questionnaire. But, the current study seems to point out a knowledge gap between nurses and doctors concerning dengue warning signs in this study: for example, more nurses (16.5%) chose “do not know” compared to physicians (3.8%) in choosing a definition for abdominal pain. This holds for most warning signs except for lethargy, which showed more physicians (29.5%) choosing “do not know” versus 25.7% for nurses. Interpretation of lethargy is still many ambiguities, and an objective differentiation from tiredness is lacking might be a possible explanation. Another possibility could be nurses having more contact with patients [21]. Interestingly, nurses and physicians were similar in frequency in choosing “do not know” concerning the definitions for the decrease in platelet count warning sign: 21.1% for physicians versus 19.9% for nurses. One of the possible explanation is HCPs including doctors and nurses have received continuous education on dengue. However, training in health facilities are more likely concentrated on treatment rather than transmission, clinical classification and diagnosis. Doctors and nurses in Vietnam, for example, are supposed to understand how to diagnose and classify dengue cases, therefore continuous education health units will most likely update new points related to treatment. Experience in terms of years in treating dengue does not seem to play that big of a role for the knowledge gap as the average mean of years for nurses is 6.7 years versus 5.3 years for physicians, according to Table 1.

Additionally, the variation amongst the definitions of the warning choices may partly be due to random guessing. Finally, we cannot comment on the geographical differences playing a role as the sample size of doctors and nurses at hospitals in Pakistan and Egypt is too small versus Vietnam, of which this study has the majority (89.8% of total sample size). Furthermore, varying in current dengue situation, public health response and clinical classification system of dengue cases in three countries might have enormous impact on the results. To improve this study, a bigger sample size overall is needed especially in hospitals of Pakistan and Egypt, in which the study can then stratify and analyze data variations by geography or hospitals.

Despite having mostly vague warning criteria, the only specific WHO 2009 classification of “liver enlargement > 2 cm” is recognized by only 57.5% by physicians and 45.3% by nurses of this study. This points out that part of the knowledge gap is not due to just the “vague” WHO 2009 criteria, but also has to do with either the HCPs’ training or something else. Instead, nearly 65% of HCPs chose painful hepatomegaly as a warning sign, and 15% of HCPs says that it depends on the case.

For certain warning signs, it is evident that when HCPs use more definitions that it would increase the overall sensitivity of the WHO 2009 criteria. For example, for the plasma leakage warning sign, the HCPs must be wary for pleural effusion, ascites, edema (face and extremities), or the hemoconcentration. Thus, it is dependent on the healthcare provider’s ability to detect more physical exam signs or abnormal lab values, which means that training, experience, and a HCPs’ knowledge bank plays a significant role in the triaging of a patient. Thus, it would be recommended that hospitals in endemic areas to have more training of HCPs in the knowledge and recognition of dengue patients, as well as have a concise clinical protocol for them to follow.

Recognition of dengue fever might be complicated because not only clinical manifestation of dengue might vary from person to person according to age or gender, but also there may be different defining criteria that vary from country to country defined and agreed upon by different experts, and maybe redefined later due to regional events. For instance, a survey conducted on primary care providers (PCPs) in Singapore presented that 52.2% of PCPs set a platelet cutoff < 80.000 mm^3^ as the threshold for hospital referral. However, after the epidemic in 2013, there was a significant increase in the PCPs who use < 50.000 mm^3^ as the cutoff. This number could even be lower, as seen in PCPs from Pakistan, as a survey from 2011 showed that 76% of them use < 20.000 mm^3^ as the platelet cutoff [22]. In our study, 43.3% of physicians, and 55.5% of nurses used < 100.000 mm^3^ as the definition criteria for the warning sign of platelet decrease (Table 3). Warning signs were determined based on laboratory tests such as hemoconcentration are more likely to identify by doctors. This may be a reason for a significantly higher proportion of nurses who unknown this sign. It may be that having not a global definition of a warning sign may be beneficial because dengue might appear differently regionally. Also, scientific studies are done regionally, which may affect the current expert consensus of what the definition of a warning sign would be for that region.

Although clinical criteria can be different for different regions, the WHO 2009 guidelines recognize the use of the NS1 strip test for early recognition and diagnosis of acute infection of dengue, and the IgM ELISA test for being specific to diagnose dengue infection. In our study, only 50.9% of HCPs were able to recognize the NS1 strip test, and 42.9% knew about the IgM ELISA as serology specific test to diagnose dengue infection. The majority of physicians preferred dengue serology test to polymerase chain reaction or NS1 antigen test [23]. The availability and affordability of serology tests may explain limited usage of the NS1 strip test. A study in Singapore reported only 11.1% of PCPs used NS1 antigen, but HCPs in private settings utilized NS1 antigen nearly ten times higher than polyclinic counterparts [24]. Our survey was conducted in public settings, where the NS1 antigen might not be affordable, which can result in a knowledge gap among HCPs. Neglecting the NS1 test may hinder the early diagnosis of dengue, as the dengue serology tests have been shown to have low specificity and may be falsely negative during the early febrile phase of illness [25, 26]. Hence, improving knowledge of physician regarding value of dengue diagnostic test is an area of potential improvement based on our findings.

Basic knowledge about dengue (the mosquito, the route of transmission) may not, in the beginning, help with the triage or the clinical management of patients but may be valuable in terms of preventive care for patients and families to avoid primary and secondary infection. In our study, 86% of HCPs chose correctly that the Aedes aegypti mosquito is the primary vector for the dengue infection; however, only 31.6% of HCPs chose correctly that dengue mosquitos are more likely to bite during the daytime. Also, few HCPs were able to identify the different routes of transmission: only 10.4% of HCPs identified mother to fetus or neonatal transmission, 7.2% for blood transfusion and organ transplantation, 3.2% for needle stick, and only 0.6% for sexual intercourse. Thus, there needs to be an improvement of HCPs’ preventive care knowledge in order to reduce dengue infection as well as the HCPs’ attitude in educating the patients and their families. In the current study, 90.2% of HCPs “agree” or “strongly agree” with their responsibility to talk to the patients’ family about dengue virus transmission and prevention. However, only 75.9% of HCPs during outpatient regularly recommend measures to prevent mosquito bites at home, and 73.1% advise on measures to prevent dengue fever for other people living at home with the patient. Hence, increasing HCPs’ knowledge of Aedes mosquito, the different routes of dengue transmission, and knowledge of the biting time of mosquitos is another area for improvement based on our survey.

Our survey has been conducted in three countries: Vietnam, Egypt, and Pakistan. In Vietnam, the dengue threat increased gradually from killing 32.5 people out of every 100.000 in 2000 to kill about 120 people out of every 100.000 in 2009. This study is valuable because Vietnam carries the highest burden of dengue infection in the Asia sub-region, especially the south of Vietnam [27, 28]. Besides, the majority of HCPs of this survey was in Vietnam, which makes this survey especially valuable. Egypt had a recent outbreak in November 2015 in the Dayrout District of Assiut Governorate [29, 30]. Pakistan experienced an epidemic of dengue fever since 2010, which so far infected 16.580 and killed 257 people in Lahore [29, 31]. Therefore, the HCPs working in those three countries should represent a sample that is the potential to deal with dengue patients, which urge the investigation of their knowledge, attitude, and practice.

Again, this study may be limited by the variation in sample size among the three countries, where we conducted our survey, with the vast majority of respondents were in Vietnam. Therefore, the results of this current study should be interpreted in the context of response bias. Lack of sample size calculation is another limitation of our study. Besides, our results should be considered the gap of demographic cofactors and the state of the dengue epidemic in each country. To improve the study later, we are increasing more investigations with a larger sample size to identify the error precisely and fix it by the healthcare authorities and organizations.

## Conclusion

The current study highlighted the wide variation in the application of all warning signs listed in WHO 2009 classification and significant gaps in knowledge about dengue diagnosis test, Aedes aegypti and dengue rout of transmission among HCPs. These results provide a valuable opportunity to identify areas of dengue management that need improvement. Interventions should focus on increasing knowledge of HCPs regarding dengue transmission, clinical classification and diagnosis.

## Supplementary Information

**Additional file 1.** Factors associated with knowledge, attitude and practice of HCPs.

## Data Availability

The datasets used and/or analysed during the current study are available from the corresponding author on reasonable request.
