# Supplementary material for: Survey of knowledge, attitude and practice of healthcare professionals on dengue transmission, diagnosis and clinical classification
Source: BMC Infect Dis. 2021 Nov 2;21:1130. doi: 10.1186/s12879-021-06816-y (PMC8564985; doi:10.1186/s12879-021-06816-y)
Supplement: Supplementary file 1 — Additional file 1. Factors associated with knowledge, attitude and practice of HCPs. [file 12879_2021_6816_MOESM1_ESM.docx]

| **Additional file Table S1. Factors associated to knowledge on dengue** | | | | |
| --- | --- | --- | --- | --- |
| Characteristics | Sufficient knowledge on dengue  n=176 | Insufficient knowledge on dengue  n=295 | OR (95% CI)  multivariate | P value |
| Physician | 79 (44.9) | 107 (36.3) | 1.53 (1.01,2.32) | 0.047 |
| Vietnamese HCPs | 167 (94.9) | 256 (86.8) | 3.17 (1.2,8.41) | 0.01 |
| Received trained on dengue | 145 (83.3) | 211 (72.8) | 1.47 (0.88,2.48) | 0.1 |
| Taking care ≥1 case/week | 86 (49.7) | 107 (37.9) | 1.35 (0.91,2.01) | 0.1 |
| Sufficient knowledge on dengue if HCPs answered correctly ≥4 questions among 5 questions (1. Mosquito that can spread dengue fever (Aedes aegypti); 2. Transmission route (human to human via a bite of an infected mosquito); 3. Time when the dengue mosquito likely to bite (day time); 4. Lifelong immunity to infected serotype (true); 5. Most common test that takes shorter time to diagnose acute recent dengue infection (NS1) | | | | |

| **Additional file Table S2. Attitude and practice towards dengue fever among 471 healthcare professionals** | | | | | |
| --- | --- | --- | --- | --- | --- |
| Category of question | | Proportion of respondents | | | |
|  |  | **Total**  (N=471)  n (%) | **Doctor** (N=186)  n (%) | **Nurse** (N=285)  n (%) | p |
| It is your responsibility to talk to the patient's family about dengue virus transmission and prevention | | **469** | **185** | **284** |  |
|  | Strongly agree | 232 (49.5) | 103 (55.7) | 129 (45.4) | 0.03 |
|  | Agree | 191 (40.7) | 67 (36.2) | 124 (43.7) | 0.1 |
|  | Neutral | 31 (6.6) | 11 (5.9) | 20 (7.0) | 0.6 |
|  | Disagree | 12 (2.6) | 4 (2.2) | 8 (2.8) | 0.7 |
|  | Strongly disagree | 3 (0.6) | 0 | 3 (1.1) |  |
| How often do talk to the patient's family about the cause of dengue hemorrhagic fever? | | **470** | **185** | **285** |  |
|  | Always | 124 (26.4) | 51 (27.5) | 73 (25.6) | 0.6 |
|  | Often | 223 (47.4) | 93 (50.3) | 130 (45.6) | 0.3 |
|  | Sometimes | 102 (21.7) | 32 (17.3) | 70 (24.6) | 0.06 |
|  | Seldom | 7 (1.5) | 4 (2.2) | 3 (1.0) | 0.3 |
|  | Never | 14 (3.0) | 5 (2.7) | 9 (3.2) | 0.8 |
| If the patient is outpatient, do you regularly recommend measures to help prevent mosquito bites in patients when they are at home? | | **469** | **184** | **285** |  |
|  | Always | 158 (33.7) | 55 (29.9) | 103 (36.1) | 0.1 |
|  | Often | 198 (42.2) | 74 (40.2) | 124 (43.5) | 0.5 |
|  | Sometimes | 90 (19.2) | 44 (23.9) | 46 (16.1) | 0.03 |
|  | Seldom | 9 (1.9) | 5 (2.7) | 4 (1.5) | 0.3 |
|  | Never | 14 (3.0) | 6 (3.3) | 8 (2.8) | 0.7 |
| Do you regularly advise on measures to help prevent dengue hemorrhagic fever for other people living in the same house as the patient? | | **469** | **184** | **285** |  |
|  | Always | 143 (30.5) | 56 (30.4) | 87 (30.5) | 0.9 |
|  | Often | 200 (42.6) | 76 (41.3) | 124 (43.6) | 0.6 |
|  | Sometimes | 90 (19.2) | 38 (20.7) | 52 (18.2) | 0.5 |
|  | Seldom | 21 (4.5) | 7 (3.8) | 14 (4.9) | 0.5 |
|  | Never | 15 (3.2) | 7 (3.8) | 8 (2.8) | 0.5 |
| Would give IV fluid or Ibuprofen for fever in dengue patients | | 47 (10.3) | 16 (8.8) | 31 (11.2) | 0.4 |

| **Additional file Table S3. Factors associated to preventive practice of HCPs** | | | | |
| --- | --- | --- | --- | --- |
| How often do talk to the patient's family about the cause of dengue hemorrhagic fever? | | | | |
| Characteristics | Always and often do | Sometimes, seldom and never | OR (95% CI)  multivariate | P value |
| How often do talk to the patient's family about the cause of dengue hemorrhagic fever? | | | | |
|  | n=347 | n=124 |  |  |
| Strongly agree and agree that it is their responsibility to talk to patients and family about dengue virus transmission and prevention | 327 (94.2) | 98 (790.0) | 4.88 (2.55,9.35) | <0.001 |
| Physician | 144 (41.5) | 42 (33.9) | 1.24 (0.75,2.06) | 0.4 |
| Vietnamese HCPs | 321 (92.5) | 102 (82.3) | 0.94 (0.36,2.4) | 0.9 |
| Received trained on dengue | 272 (80.0) | 84 (67.7) | 1.19 (0.68,2.07) | 0.5 |
| Taking care ≥1 case/week | 156 (45.1) | 37 (33.9) | 1.58 (0.98,2.55) | 0.055 |
| 18. If the patient is outpatient, do you regularly recommend measures to help prevent mosquito bites in patients when they are at home? | | | | |
|  | n=356 | n=115 |  |  |
| Strongly agree and agree that it is their responsibility to talk to patients and family about dengue virus transmission and prevention | 334 (94.8) | 91 (79.1) | 5.25 (2.71,10.18) | <0.001 |
| Physician | 129 (36.2) | 57 (49.6) | 0.51 (0.31,0.84) | 0.009 |
| Vietnamese HCPs | 330 (92.7) | 93 (80.9) | 0.75 (0.29,1.9) | 0.5 |
| Received trained on dengue | 275 (77.9) | 81 (73.0) | 0.75 (0.4,1.42) | 0.3 |
| Taking care ≥1 case/week | 158 (44.8) | 35 (34.3) | 1.59 (0.97,2.63) | 0.065 |
| 20. Do you regularly advise on measures to help prevent dengue hemorrhagic fever for other people living in the same house as the patient? | | | | |
|  | n=343 | n=128 |  |  |
| Strongly agree and agree that it is their responsibility to talk to patients and family about dengue virus transmission and prevention | 316 (92.1) | 109 (85.2) | 2.49 (1.28,4.84) | 0.009 |
| Physician | 132 (38.5) | 54 (42.2) | 0.72 (0.45,1.15) | 0.1 |
| Vietnamese HCPs | 312 (91.0) | 111 (86.7) | 0.33 (0.11,0.97) | 0.03 |
| Received trained on dengue | 260 (76.7) | 96 (76.8) | 0.61 (0.33,1.13) | 0.1 |
| Taking care ≥1 case/week | 157 (46.0) | 36 (31.6) | 2.12 (1.32,3.39) | 0.001 |

| **Additional file Table S4. Variation in definition of each sign** | | | | | |
| --- | --- | --- | --- | --- | --- |
| Signs | | **Yes number (%)** | Univariate analysis | Multivariate analysis | |
| **Abdominal pain** | | **Abdominal tenderness** | OR (95% CI) | OR (95% CI) | P value |
| Professional | **Doctor** | 85 (45.7) | 1.72 (1.17,2.53) | 1.37 (0.9,2.1) | 0.1 |
|  | Nurse | 93 (32.6) |  |  |  |
| Country | **Vietnam** | 156 (36.9) | 0.47 (0.23,0.96) | 0.36 (0.16,0.83) | 0.015 |
|  | Not Vietnam | 22 (45.8) |  |  |  |
| Training | **Train** | 152 (42.7) | 2.42 (1.43,4.07) | 2.8 (1.59,4.93) | <0.001 |
|  | Not train | 25 (23.1) |  |  |  |
| Frequency of taking care dengue patients | **Often very often** | 78 (40.4) | 1.16 (0.79,1.7) | 1.25 (0.84,1.88) | 0.2 |
|  | Seldom | 97 (37.0) |  |  |  |
|  | | **Continuous abdominal paint** |  |  |  |
| Professional | Doctor | 93 (50.0) | 1.48 (1.01,2.17) | 1.91 (1.26,2.9) | 0.002 |
|  | Nurse | 107 (37.5) |  |  |  |
| Country | Vietnam | 190 (44.9) | 6 (2.08,17.34) | 7.81 (2.54,24) | <0.001 |
|  | Not Vietnam | 10 (20.8) |  |  |  |
| Training | Train | 163 (45.8) | 2.31 (1.4,3.82) | 1.78 (1.06,3.02) | 0.03 |
|  | Not train | 31 (28.7) |  |  |  |
| Frequency of taking care dengue patients | Often very often | 87 (45.1) | 1.2 (0.82,1.75) | 0.97 (0.65,1.44) | 0.8 |
|  | Seldom | 107 (40.8) |  |  |  |
|  |  | **Increasing pain** |  |  |  |
| Professional | Doctor | 70 (37.6) | 0.71 (0.49,1.05) | 0.87 (0.58,1.32) | 0.5 |
|  | Nurse | 132 (46.3) |  |  |  |
| Country | Vietnam | 194 (45.9) | 4.97 (1.89,13.08) | 4.54 (1.62,12.76) | 0.001 |
|  | Not Vietnam | 8 (16.7) |  |  |  |
| Training | Train | 163 (45.8) | 1.46 (0.91,2.33) | 1.28 (0.78,2.1) | 0.3 |
|  | Not train | 38 (35.2) |  |  |  |
| Frequency of taking care dengue patients | Often very often | 83 (43.0) | 0.98 (0.67,1.43) | 0.85 (0.57,1.25) | 0.4 |
|  | Seldom | 114 (43.5) |  |  |  |
|  |  | **Do not know/Not sure** |  |  |  |
| Professional | Doctor | 7 (3.8) | 0.24 (0.1,0.54) | 0.27 (0.11,0.65) | 0.001 |
|  | Nurse | 47 (16.5) |  |  |  |
| Country | Vietnam | 47 (11.1) | 4.12 (0.55,30.88) | 1.7 (0.19,15.13) | 0.6 |
|  | Not Vietnam | 7 (14.6) |  |  |  |
| Training | Train | 30 (8.4) | 0.41 (0.22,0.79) | 0.42 (0.21,0.83) | 0.015 |
|  | Not train | 23 (21.3) |  |  |  |
| Frequency of taking care dengue patients | Often very often | 27 (14.0) | 1.95 (1.06,3.6) | 2.02 (1.07,3.82) | 0.03 |
|  | Seldom | 21 (8.0) |  |  |  |
| **Persistent vomiting** | | **≥ 6 episodes of vomiting in 24 hours** |  |  |  |
| Professional | Doctor | 37 (19.9) | 1.08 (0.67,1.74) | 0.98 (0.58,1.64) | 0.9 |
|  | Nurse | 51 (17.9) |  |  |  |
| Country | Vietnam | 79 (18.7) | 0.9 (0.38,2.13) | 0.68 (0.25,1.84) | 0.4 |
|  | Not Vietnam | 9 (18.8) |  |  |  |
| Training | Train | 72 (20.2) | 1.57 (0.83,2.97) | 1.63 (0.84,3.17) | 0.1 |
|  | Not train | 15 (13.9) |  |  |  |
| Frequency of taking care dengue patients | Often very often | 40 (20.7) | 1.25 (0.78,2.01) | 1.28 (0.78,2.1) | 0.3 |
|  | Seldom | 46 (17.6) |  |  |  |
|  |  | **≥ 5 episodes of vomiting in 24 hours** |  |  |  |
| Professional | Doctor | 29 (15.6) | 1.61 (0.91,2.85) | 1.39 (0.75,2.6) | 0.3 |
|  | Nurse | 28 (9.8) |  |  |  |
| Country | Vietnam | 50 (11.8) | 0.49 (0.2,1.2) | 0.5 (0.18,1.42) | 0.2 |
|  | Not Vietnam | 7 (14.6) |  |  |  |
| Training | Train | 43 (12.1) | 1.03 (0.51,2.08) | 1.1 (0.52,2.31) | 0.8 |
|  | Not train | 11 (10.2) |  |  |  |
| Frequency of taking care dengue patients | Often very often | 26 (13.5) | 1.29 (0.73,2.28) | 1.43 (0.78,2.61) | 0.2 |
|  | Seldom | 31 (11.8) |  |  |  |
|  |  | **≥ 3 episodes of vomiting in 1 hour** |  |  |  |
| Professional | Doctor | 52 (28.0) | 0.99 (0.65,1.5) | 1.04 (0.66,1.62) | 0.8 |
|  | Nurse | 82 (28.8) |  |  |  |
| Country | Vietnam | 130 (30.7) | 7.08 (1.67,29.99) | 4.35 (0.96,19.66) | 0.2 |
|  | Not Vietnam | 4 (8.3) |  |  |  |
| Training | Train | 120 (33.7) | 4.77 (2.32,9.81) | 4.21 (2.02,8.79) | <0.001 |
|  | Not train | 11 (10.2) |  |  |  |
| Frequency of taking care dengue patients | Often very often | 69 (35.8) | 1.78 (1.18,2.68) | 1.57 (1.02,2.41) | 0.04 |
|  | Seldom | 63 (24.0) |  |  |  |
|  |  | **Vomiting for ≥2 consecutive days** |  |  |  |
| Professional | Doctor | 46 (27.4) | 0.59 (0.39,0.9) | 0.74 (0.48,1.15) | 0.2 |
|  | Nurse | 103 (36.1) |  |  |  |
| Country | Vietnam | 145 (34.3) | 5.45 (1.64,18.14) | 5.29 (1.5,18.72) | 0.002 |
|  | Not Vietnam | 4 (8.3) |  |  |  |
| Training | Train | 117 (32.9) | 1.08 (0.66,1.77) | 0.98 (0.58,1.64) | 0.9 |
|  | Not train | 30 (27.8) |  |  |  |
| Frequency of taking care dengue patients | Often very often | 57 (29.5) | 0.77 (0.51,1.15) | 0.68 (0.45,1.03) | 0.07 |
|  | Seldom | 91 (34.7) |  |  |  |
|  |  | **Vomiting with signs of dehydration on physical examination** |  |  |  |
| Professional | Doctor | 55 (29.6) | 1.07 (0.71,1.63) | 0.93 (0.58,1.47) | 0.7 |
|  | Nurse | 79 (27.7) |  |  |  |
| Country | Vietnam | 116 (27.4) | 0.54 (0.26,1.1) | 0.45 (0.2,1.03) | 0.06 |
|  | Not Vietnam | 18 (37.5) |  |  |  |
| Training | Train | 97 (27.2) | 0.74 (0.45,1.21) | 0.81 (0.48,1.35) | 0.4 |
|  | Not train | 36 (33.3) |  |  |  |
| Frequency of taking care dengue patients | Often very often | 62 (32.1) | 1.42 (0.94,2.15) | 1.6 (1.04,2.46) | 0.03 |
|  | Seldom | 66 (25.2) |  |  |  |
|  |  | **Vomiting whenever eating/drinking** |  |  |  |
| Professional | Doctor | 45 (24.2) | 1.32 (0.84,2.07) | 1.51 (0.91,2.5) | 0.1 |
|  | Nurse | 55 (19.3) |  |  |  |
| Country | Vietnam | 95 (22.5) | 1.73 (0.65,4.59) | 1.45 (0.47,4.45) | 0.5 |
|  | Not Vietnam | 5 (10.4) |  |  |  |
| Training | Train | 73 (20.5) | 0.63 (0.38,1.06) | 0.49 (0.28,0.87) | 0.02 |
|  | Not train | 27 (25.0) |  |  |  |
| Frequency of taking care dengue patients | Often very often | 70 (36.3) | 4.38 (2.71,7.08) | 4.44 (2.68,7.35) | <0.001 |
|  | Seldom | 30 (11.5) |  |  |  |
|  |  | **Do not know/Not sure** |  |  |  |
| Professional | Doctor | 17 (9.1) | 0.71 (0.38,1.33) | 0.86 (0.43,1.72) | 0.7 |
|  | Nurse | 40 (14.0) |  |  |  |
| Country | Vietnam | 47 (11.1) | 1.29 (0.38,4.39) | 1.9 (0.48,7.48) | 0.3 |
|  | Not Vietnam | 10 (20.8) |  |  |  |
| Training | Train | 31 (8.7) | 0.4 (0.21,0.75) | 0.38 (0.2,0.74) | 0.006 |
|  | Not train | 25 (23.1) |  |  |  |
| Frequency of taking care dengue patients | Often very often | 17 (8.8) | 0.69 (0.37,1.28) | 0.67 (0.35,1.27) | 0.2 |
|  | Seldom | 33 (12.6) |  |  |  |
| **Mucosal bleeding** | | **Epistaxis** |  |  |  |
| Professional | Doctor | 149 (80.1) | 1.55 (0.99,2.42) | 2.89 (1.6,5.23) | < 0.001 |
|  | Nurse | 199 (69.8) |  |  |  |
| Country | Vietnam | 325 (76.8) | 6.33 (3.02,13.27) | 8.56 (3.45,21.25) | < 0.001 |
|  | Not Vietnam | 23 (47.9) |  |  |  |
| Training | Train | 278 (78.1) | 2.49 (1.54,4.05) | 1.8 (1.06,3.06) | 0.03 |
|  | Not train | 66 (61.1) |  |  |  |
| Frequency of taking care dengue patients | Often very often | 164 (85.0) | 2.98 (1.85,4.8) | 2.39 (1.45,3.96) | < 0.001 |
|  | Seldom | 173 (66.0) |  |  |  |
|  |  | **Gingival bleeding** |  |  |  |
| Professional | Doctor | 149 (80.1) | 0.75 (0.46,1.23) | 1.22 (0.66,2.26) | 0.5 |
|  | Nurse | 237 (83.2) |  |  |  |
| Country | Vietnam | 362 (85.6) | 7.77 (3.74,16.16) | 6.23 (2.55,15.22) | < 0.001 |
|  | Not Vietnam | 24 (50.0) |  |  |  |
| Training | Train | 306 (86.0) | 2.5 (1.46,4.28) | 1.86 (1.03,3.35) | 0.04 |
|  | Not train | 76 (70.4) |  |  |  |
| Frequency of taking care dengue patients | Often very often | 171 (88.6) | 2.26 (1.31,3.88) | 1.69 (0.95,2.99) | 0.07 |
|  | Seldom | 204 (77.9) |  |  |  |
|  |  | **Hematemesis, Melena** |  |  |  |
| Professional | Doctor | 123 (66.1) | 1.26 (0.85,1.87) | 1.63 (1.04,2.55) | 0.03 |
|  | Nurse | 172 (60.4) |  |  |  |
| Country | Vietnam | 274 (64.8) | 3.88 (1.84,8.18) | 3.98 (1.71,9.25) | < 0.001 |
|  | Not Vietnam | 21 (43.8) |  |  |  |
| Training | Train | 234 (65.7) | 1.98 (1.24,3.14) | 1.58 (0.97,2.57) | 0.07 |
|  | Not train | 56 (51.9) |  |  |  |
| Frequency of taking care dengue patients | Often very often | 135 (69.9) | 1.79 (1.2,2.66) | 1.51 (1,2.29) | 0.048 |
|  | Seldom | 150 (57.3) |  |  |  |
|  |  | **Hemoptysis** |  |  |  |
| Professional | Doctor | 58 (31.2) | 2.3 (1.46,3.6) | 2.54 (1.57,4.12) | < 0.001 |
|  | Nurse | 46 (16.1) |  |  |  |
| Country | Vietnam | 97 (22.9) | 1.13 (0.48,2.68) | 1.66 (0.64,4.36) | 0.3 |
|  | Not Vietnam | 7 (14.6) |  |  |  |
| Training | Train | 80 (22.5) | 1 (0.58,1.72) | 0.8 (0.45,1.43) | 0.5 |
|  | Not train | 21 (19.4) |  |  |  |
| Frequency of taking care dengue patients | Often very often | 54 (28.0) | 1.76 (1.13,2.75) | 1.68 (1.05,2.68) | 0.03 |
|  | Seldom | 50 (19.1) |  |  |  |
|  |  | **Ear bleeding** |  |  |  |
| Professional | Doctor | 42 (22.6) | 1.88 (1.14,3.11) | 2.17 (1.27,3.73) | 0.005 |
|  | Nurse | 36 (12.6) |  |  |  |
| Country | Vietnam | 72 (17.0) | 1.16 (0.43,3.1) | 1.81 (0.6,5.43) | 0.3 |
|  | Not Vietnam | 6 (12.5) |  |  |  |
| Training | Train | 55 (15.4) | 0.71 (0.4,1.28) | 0.58 (0.31,1.07) | 0.08 |
|  | Not train | 20 (18.5) |  |  |  |
| Frequency of taking care dengue patients | Often very often | 39 (20.2) | 1.63 (0.99,2.69) | 1.56 (0.92,2.62) | 0.09 |
|  | Seldom | 38 (14.5) |  |  |  |
|  |  | **Hematuria** |  |  |  |
| Professional | Doctor | 94 (50.5) | 3.15 (2.1,4.73) | 4.23 (2.7,6.6) | < 0.001 |
|  | Nurse | 72 (25.3) |  |  |  |
| Country | Vietnam | 150 (35.5) | 1.77 (0.78,4.01) | 3.95 (1.58,9.9) | 0.002 |
|  | Not Vietnam | 16 (33.3) |  |  |  |
| Training | Train | 120 (33.7) | 0.89 (0.55,1.43) | 0.59 (0.35,0.99) | 0.048 |
|  | Not train | 42 (38.9) |  |  |  |
| Frequency of taking care dengue patients | Often very often | 82 (42.5) | 1.93 (1.3,2.87) | 1.76 (1.15,2.7) | 0.009 |
|  | Seldom | 76 (29.0) |  |  |  |
|  |  | **Menorrhagia, metrorrhagia, vaginal bleeding** |  |  |  |
| Professional | Doctor | 116 (62.4) | 1.39 (0.94,2.05) | 2.09 (1.34,3.27) | < 0.001 |
|  | Nurse | 152 (53.3) |  |  |  |
| Country | Vietnam | 261 (61.7) | 6.25 (2.66,14.69) | 8.2 (3.19,21.11) | < 0.001 |
|  | Not Vietnam | 7 (14.6) |  |  |  |
| Training | Train | 214 (60.1) | 1.36 (0.86,2.16) | 0.96 (0.58,1.58) | 0.8 |
|  | Not train | 49 (45.4) |  |  |  |
| Frequency of taking care dengue patients | Often very often | 134 (69.4) | 2.24 (1.51,3.32) | 1.83 (1.22,2.76) | 0.003 |
|  | Seldom | 134 (51.1) |  |  |  |
|  |  | **Petechia, purpura, ecchymosis, bruises** |  |  |  |
| Professional | Doctor | 72 (38.7) | 0.49 (0.33,0.73) | 0.45 (0.29,0.68) | < 0.001 |
|  | Nurse | 154 (54.0) |  |  |  |
| Country | Vietnam | 199 (47.0) | 0.7 (0.34,1.41) | 0.55 (0.25,1.23) | 0.1 |
|  | Not Vietnam | 27 (56.2) |  |  |  |
| Training | Train | 158 (44.4) | 0.55 (0.35,0.88) | 0.65 (0.4,1.05) | 0.08 |
|  | Not train | 63 (58.3) |  |  |  |
| Frequency of taking care dengue patients | Often very often | 80 (41.5) | 0.65 (0.44,0.94) | 0.7 (0.47,1.04) | 0.07 |
|  | Seldom | 138 (52.7) |  |  |  |
|  |  | **Conjunctival, subconjunctival, retinal hemmorrhage** |  |  |  |
| Professional | Doctor | 108 (58.1) | 1.46 (1,2.13) | 1.78 (1.17,2.72) | 0.007 |
|  | Nurse | 138 (48.4) |  |  |  |
| Country | Vietnam | 226 (53.4) | 2.12 (1.02,4.4) | 2.57 (1.12,5.88) | 0.02 |
|  | Not Vietnam | 20 (41.7) |  |  |  |
| Training | Train | 184 (51.7) | 0.93 (0.59,1.46) | 0.73 (0.45,1.2) | 0.2 |
|  | Not train | 58 (53.7) |  |  |  |
| Frequency of taking care dengue patients | Often very often | 121 (62.7) | 2.2 (1.5,3.23) | 2.02 (1.36,3.01) | < 0.001 |
|  | Seldom | 117 (44.7) |  |  |  |
|  |  | **Do not know/Not sure** |  |  |  |
| Professional | Doctor | 4 (2.2) | 2.05 (0.45,9.25) | 1.92 (0.31,11.74) | 0.5 |
|  | Nurse | 4 (1.4) |  |  |  |
| Country | Vietnam | 5 (1.2) | 0.2 (0.04,1.05) | 0.68 (0.08,5.72) | 0.7 |
|  | Not Vietnam | 3 (6.2) |  |  |  |
| Training | Train | 3 (0.8) | 0.19 (0.04,0.86) | 0.21 (0.04,1.07) | 0.06 |
|  | Not train | 5 (4.6) |  |  |  |
| Frequency of taking care dengue patients | Often very often | 1 (0.5) | 0.22 (0.03,1.84) | 0.27 (0.03,2.37) | 0.2 |
|  | Seldom | 6 (2.3) |  |  |  |
| **Plasma leakage** | | **Hemoconcentration** |  |  |  |
| Professional | Doctor | 162 (87.1) | 1.64 (0.97,2.77) | 2.64 (1.35,5.16) | 0.002 |
|  | Nurse | 218 (76.5) |  |  |  |
| Country | Vietnam | 354 (83.7) | 3.26 (1.56,6.84) | 5.1 (1.95,13.32) | < 0.001 |
|  | Not Vietnam | 26 (54.2) |  |  |  |
| Training | Train | 302 (84.8) | 2.21 (1.29,3.79) | 1.67 (0.95,2.94) | 0.08 |
|  | Not train | 73 (67.6) |  |  |  |
| Frequency of taking care dengue patients | Often very often | 165 (85.5) | 1.54 (0.93,2.56) | 1.24 (0.73,2.13) | 0.4 |
|  | Seldom | 209 (79.8) |  |  |  |
|  |  | **Pleural effusion** |  |  |  |
| Professional | Doctor | 130 (69.9) | 1.84 (1.23,2.74) | 2.53 (1.58,4.03) | < 0.001 |
|  | Nurse | 157 (55.1) |  |  |  |
| Country | Vietnam | 266 (62.9) | 3.64 (1.73,7.68) | 4.85 (2.06,11.43) | < 0.001 |
|  | Not Vietnam | 21 (43.8) |  |  |  |
| Training | Train | 234 (65.7) | 2.56 (1.61,4.08) | 1.98 (1.21,3.23) | 0.006 |
|  | Not train | 50 (46.3) |  |  |  |
| Frequency of taking care dengue patients | Often very often | 132 (68.4) | 1.74 (1.18,2.58) | 1.46 (0.96,2.21) | 0.08 |
|  | Seldom | **145 (55.3)** |  |  |  |
|  |  | **Gall bladder thickening** |  |  |  |
| Professional | Doctor | 95 (51.1) | 3.51 (2.34,5.28) | 3.93 (2.54,6.1) | < 0.001 |
|  | Nurse | 64 (22.5) |  |  |  |
| Country | Vietnam | 148 (35.0) | 1.29 (0.6,2.78) | 2.15 (0.91,5.07) | 0.08 |
|  | Not Vietnam | 11 (22.9) |  |  |  |
| Training | Train | 135 (37.9) | 2.24 (1.31,3.84) | 1.79 (1.01,3.18) | 0.04 |
|  | Not train | 21 (19.4) |  |  |  |
| Frequency of taking care dengue patients | Often very often | 76 (39.4) | 1.49 (1.01,2.21) | 1.35 (0.88,2.07) | 0.2 |
|  | Seldom | 82 (31.3) |  |  |  |
|  |  | **Ascites** |  |  |  |
| Professional | Doctor | 101 (54.3) | 2.29 (1.55,3.38) | 2.66 (1.74,4.06) | < 0.001 |
|  | Nurse | 97 (34.0) |  |  |  |
| Country | Vietnam | 177 (41.8) | 1.76 (0.82,3.77) | 2.45 (1.04,5.72) | 0.03 |
|  | Not Vietnam | 21 (43.8) |  |  |  |
| Training | Train | 155 (43.5) | 1.63 (1,2.64) | 1.3 (0.78,2.16) | 0.3 |
|  | Not train | 41 (38.0) |  |  |  |
| Frequency of taking care dengue patients | Often very often | 94 (48.7) | 1.77 (1.21,2.59) | 1.61 (1.08,2.4) | 0.02 |
|  | Seldom | 93 (35.5) |  |  |  |
|  |  | **Edema (face and extremities)** |  |  |  |
| Professional | Doctor | 64 (34.4) | 1.74 (1.14,2.66) | 1.93 (1.22,3.05) | 0.005 |
|  | Nurse | 66 (23.2) |  |  |  |
| Country | Vietnam | 114 (27.0) | 0.88 (0.41,1.9) | 1.32 (0.55,3.17) | 0.5 |
|  | Not Vietnam | 16 (33.3) |  |  |  |
| Training | Train | 89 (25.0) | 0.64 (0.39,1.04) | 0.55 (0.33,0.92) | 0.026 |
|  | Not train | 38 (35.2) |  |  |  |
| Frequency of taking care dengue patients | Often very often | 62 (32.1) | 1.54 (1.01,2.34) | 1.54 (0.99,2.39) | 0.053 |
|  | Seldom | 62 (23.7) |  |  |  |
|  |  | **Free fluids around urinary bladder** |  |  |  |
| Professional | Doctor |  | 1.31 (0.84,2.04) | 1.37 (0.85,2.22) | 0.2 |
|  | Nurse |  |  |  |  |
| Country | Vietnam |  | 1.47 (0.59,3.64) | 1.25 (0.45,3.46) | 0.7 |
|  | Not Vietnam |  |  |  |  |
| Training | Train |  | 0.98 (0.58,1.69) | 0.87 (0.49,1.53) | 0.6 |
|  | Not train |  |  |  |  |
| Frequency of taking care dengue patients | Often very often |  | 2.52 (1.61,3.94) | 2.47 (1.56,3.92) | < 0.001 |
|  | Seldom |  |  |  |  |
|  |  | **Do not know/Not sure** |  |  |  |
| Professional | Doctor | 4 (2.2) | 0.24 (0.08,0.69) | 0.13 (0.03,0.6) | < 0.001 |
|  | Nurse | 26 (9.1) |  |  |  |
| Country | Vietnam | 25 (5.9) | 0.66 (0.19,2.32) | 0.2 (0.03,1.36) | 0.09 |
|  | Not Vietnam | 5 (10.4) |  |  |  |
| Training | Train | 16 (4.5) | 0.32 (0.15,0.7) | 0.4 (0.18,0.91) | 0.03 |
|  | Not train | 14 (13.0) |  |  |  |
| Frequency of taking care dengue patients | Often very often | 10 (5.2) | 0.73 (0.33,1.63) | 0.84 (0.36,1.93) | 0.7 |
|  | Seldom | 18 (6.9) |  |  |  |
| **Liver enlargement** | | **Liver edge palpated more than 2 cm below the costal margin** |  |  |  |
| Professional | Doctor | 107 (57.5) | 1.62 (1.1,2.37) | 1.65 (1.07,2.52) | 0.02 |
|  | Nurse | 129 (45.3) |  |  |  |
| Country | Vietnam | 213 (50.4) | 1.17 (0.58,2.36) | 1.03 (0.46,2.28) | 0.9 |
|  | Not Vietnam | 23 (47.9) |  |  |  |
| Training | Train | 184 (51.7) | 1.25 (0.79,1.98) | 1.13 (0.69,1.85) | 0.6 |
|  | Not train | 50 (46.3) |  |  |  |
| Frequency of taking care dengue patients | Often very often | 125 (64.8) | 2.77 (1.88,4.08) | 2.77 (1.86,4.14) | < 0.001 |
|  | Seldom | 104 (39.7) |  |  |  |
|  |  | **Liver edge palpated below the costal margin** |  |  |  |
| Professional | Doctor | 33 (17.7) | 0.55 (0.35,0.87) | 0.56 (0.34,0.92) | 0.02 |
|  | Nurse | 81 (28.4) |  |  |  |
| Country | Vietnam | 109 (25.8) | 2.02 (0.76,5.36) | 1.3 (0.45,3.8) | 0.6 |
|  | Not Vietnam | 5 (10.4) |  |  |  |
| Training | Train | 94 (26.4) | 1.5 (0.85,2.64) | 1.56 (0.87,2.8) | 0.1 |
|  | Not train | 18 (16.7) |  |  |  |
| Frequency of taking care dengue patients | Often very often | 45 (23.3) | 0.87 (0.57,1.35) | 0.85 (0.54,1.32) | 0.5 |
|  | Seldom | 69 (26.3) |  |  |  |
|  |  | **Painful hepatomegaly** |  |  |  |
| Professional | Doctor | 120 (64.5) | 1.51 (1.03,2.23) | 1.95 (1.26,3.02) | 0.002 |
|  | Nurse | 151 (53.0) |  |  |  |
| Country | Vietnam | 254 (60.0) | 3.16 (1.5,6.65) | 4.13 (1.78,9.6) | < 0.001 |
|  | Not Vietnam | 17 (35.4) |  |  |  |
| Training | Train | 220 (61.8) | 2.04 (1.29,3.24) | 1.63 (1,2.63) | 0.048 |
|  | Not train | 48 (44.4) |  |  |  |
| Frequency of taking care dengue patients | Often very often | 118 (61.1) | 1.26 (0.86,1.85) | 1.05 (0.71,1.57) | 0.8 |
|  | Seldom | 145 (55.3) |  |  |  |
|  |  | **Depend on each case** |  |  |  |
| Professional | Doctor | 27 (14.5) | 0.52 (0.32,0.85) | 0.54 (0.32,0.91) | 0.02 |
|  | Nurse | 70 (24.6) |  |  |  |
| Country | Vietnam | 94 (22.2) | 4.7 (1.11,19.98) | 2.17 (0.47,10.01) | 0.3 |
|  | Not Vietnam | 3 (6.2) |  |  |  |
| Training | Train | 80 (22.5) | 1.4 (0.77,2.53) | 1.37 (0.74,2.55) | 0.3 |
|  | Not train | 17 (15.7) |  |  |  |
| Frequency of taking care dengue patients | Often very often | 54 (28.0) | 2.02 (1.28,3.19) | 1.94 (1.21,3.1) | 0.005 |
|  | Seldom | 42 (16.0) |  |  |  |
|  |  | **No specific criteria** |  |  |  |
| Professional | Doctor | 6 (3.2) | 0.91 (0.32,2.54) | 0.97 (0.32,2.94) | 0.9 |
|  | Nurse | 10 (3.5) |  |  |  |
| Country | Vietnam | 15 (3.5) | 1.24 (0.16,9.69) | 1.32 (0.14,12.89) | 0.8 |
|  | Not Vietnam | 1 (2.1) |  |  |  |
| Training | Train | 12 (3.4) | 0.78 (0.25,2.47) | 0.75 (0.23,2.49) | 0.6 |
|  | Not train | 4 (3.7) |  |  |  |
| Frequency of taking care dengue patients | Often very often | 7 (3.6) | 1.05 (0.38,2.87) | 1.03 (0.37,2.89) | 0.9 |
|  | Seldom | 9 (3.4) |  |  |  |
|  |  | **Do not know/Not sure** |  |  |  |
| Professional | Doctor |  | 0.21 (0.08,0.56) | 0.1 (0.02,0.45) | < 0.001 |
|  | Nurse |  |  |  |  |
| Country | Vietnam |  | 0.65 (0.22,1.96) | 0.15 (0.02,0.9) | 0.03 |
|  | Not Vietnam |  |  |  |  |
| Training | Train |  | 0.27 (0.13,0.53) | 0.34 (0.16,0.7) | 0.004 |
|  | Not train |  |  |  |  |
| Frequency of taking care dengue patients | Often very often |  | 1.03 (0.52,2.03) | 1.23 (0.59,2.53) | 0.6 |
|  | Seldom |  |  |  |  |
| **Hemoconcentration** | |  |  |  |  |
|  | Increasing in hematocrit by more than 20% from the baseline (1) | 291 (63.5) | 2.01 (0.54,7.53) | 2.27 (0.5,10.35) |  |
|  | Increasing in hematocrit by more than 15% from the baseline (2) | 40 (8.7) |  |  |  |
|  | Using a cutoff value for hematocrit that is adjusted for gender (3) | 23 (5) |  |  |  |
|  | Using a non-adjusted cutoff value of hematocrit > 48%, with no discrimination between males and females (4) | 35 (7.6) |  |  |  |
|  | Do not know/Not sure (5) | 69 (15.1) |  |  |  |
| **Rapid decrease in platelet count** | |  |  |  |  |
|  | <20.000 (1) | 36 (8.0) |  |  |  |
|  | <50.000 (2) | (17.5) |  |  |  |
|  | <100.000 (3) | 229 (50.7) |  |  |  |
|  | <150.000 (4) | 16 (3.5) |  |  |  |
|  | Do not know/Not sure (5) | 92 (20.4) |  |  |  |
| **Lethargy** | |  |  |  |  |
|  | Alteration of consciousness and/or Glasgow score < 15 or Blantyre score less than 5 (1) | 88 (19.0) |  |  |  |
|  | Drowsiness and/or irritability (2) | 249 (53.8) |  |  |  |
|  | Do not know/Not sure (3) | 126 (27.2) |  |  |  |
|  | |  |  |  |  |
